# An Ishmaelite

**Published:** 1888-08-18

**Authors:** Leslie Keith

**Affiliations:** Author of "The Chilcotes," "Uncle Bob's Niece," "East and West," etc.


### An Ishmaelite.

By Leslie Keith,

Author of "The Chilcotes," "Uncle Bob's Niece," "East and West," etc.

(Continued from p. 314.)

Chapter XIX.—Porcelain v. Delf.

In the wintry days when the elderly Mr. Penfold was doing his lover's part and Miss Ella Coates was preparing for matrimony, Arthur Eastgate was spending his time very much to his own satisfaction.

Relieved from the necessity of listening to Ella's tinkling music and her very proper and sensible remarks, his hours were his to spend as he would. He was far kinder now to Ella than he had found it easy to be in the last months of his difficult courtship for Ella as a cousin was all he could wish. He executed her commissions with the greatest good humour, and played the part of go-between in the most amiable manner, carrying notes and messages to his successor without suffering a single pang of jealousy. But his evenings he claimed for himself, and he gave them to Bermondsey.

It is not exactly the quarter one would suppose a young man, who has all the resources of the west at his command, would be likely to choose; the busy hive on the other side of the river is a depressing enough region to most people. South London is to have its people's palaces some day—its centres of sweetness and light—but as yet it has them not. Two nations are we of London, truly, the nation that works is here, and on the other side of the broad dividing waters is the nation that plays.

What Richard Bellynge called Arthur's "philanthropic fads" did not take him to the lower deeps that are to be found here too. Gillespie Street is eminently respectable; it lies aloof from the fringe of vice, and the worst poverty does not touch it. Possibly if it had been squalid it might have had an aspect of picturesqueness for those who make copy of other people's tragedies, but being respectable it was only vulgar.

The life that went on in the tall, shabby houses was a queer parody of the society he knew best—the society of West-end drawing-rooms, where young folks play elegantly at life, uttering pretty sentiments prettily, and where it is the business of young gentlemen to analyse the ethereal moods of young ladies. Here the business of man and maid are much the same, but it was expressed with a cockney twang and without elegance; the flirtations went on more openly—in the street very often, where lads and lasses kept company on summer evenings; from an open window sometimes, where a Miss Juliet Green would thrust a head adorned with curl-papers, and exchange repartees with the handsome, curled Romeo of the barber's shop.

The girls had the same little arts and devices for making themselves fair. The ribbons were gaudier and the jewels were sham ones; but the hearts under the bright ribbons beat to pretty much the same emotions, and though the family lived and ate in one room, and practised few refinements, their aims and purposes, and the standards they raised, were not very different from those of gentlefolks of much higher degree whom he had known.

Arthur ought perhaps to have been disgusted, but he was only amused, and presently made quite a renown for himself in the neighbourhood. He was as gay and free as possible. He had his hair cut at the barber's, he had a laugh and a jesting word for Miss Maria and Miss Bell Green, and even went upstairs to their living room and listened to them as they tortured the poor old worn-out piano; he astonished his prim Cousin Ella by sending home a whole kitchenful of pots and pans and kettles from the tinsmith's, as a useful contribution towards her housekeeping; in a word, he was deep in the good graces of the whole street, and though he was rich enough to have been their enemy, he was the friend of everybody.

Even Fred was compelled to laugh at his sallies sometimes and the grim Miss Betsy surrendered to him, and showed him an amount of favour that nearly drove poor little Tucker mad.

What was it that had changed Arthur so? Was it only that he was freed from an engagement that he had felt to be a mistake? Why was it that when he entered the long low room—the comfortable, ugly room, always haunted with a fragrance from the cookshop below, and dull and dingy as a room must needs be in this close-packed region—he should yet feel himself in an enchanted atmosphere, a rainbow-coloured world? It was a pair of bright eyes and modest, blushing cheeks that did it. In the light of these, Proudie's homely ways and Mrs. Proudie's grammatical transgressions were as nothing, and indeed they were not very difficult to bear, because those simple folks made no pretence of being other than they were, and vulgarity only begins where there is an aping of gentility.

Arthur no longer urged the colonial scheme with the old fervency, or if he still dreamed of his Utopia, it was now a Utopia of three.

Fred was not slow to notice this, and one day he spoke.

"Arthur," he said, "I have let you alone for a while because it was useless to speak; but I mean to make one more effort. For—for the sake of others, you must stop coming here."

"Still harping on the old theme?" said Arthur lightly. "You are a man of one idea, Fred. How often must I tell you that I don't mean to give you up?"

"Is it me you come for, Arthur?" Fred looked at him steadily. "It was me you came for in the beginning, and I think you love me still, and would come to see me wherever I was; but it is someone else you come for now —— "

"What?" said Arthur, turning very red and laughing, "Have you found out my secret, Fred? And what if I should come at the same time to see my dearest friend and my —my—— "

"Arthur," broke out Fred vehemently, "I, of all men, have no right to turn moralist and preach to you, heaven knows, but I can't stand by and see you play with a pure and innocent girl's heart, and steal it, maybe only to throw it aside and break it. I have fallen myself, and perhaps you will resent a word of warning from me, but my own dishonour does not shut my eyes, Arthur, it only opens them more clearly, and I will save you if I can."

Arthur had looked at his cousin with a very fierce and haughty anger at the beginning of this speech, which it cost Fred some effort to make, but his earnestness and his sadness touched a gentler chord in his cousin, and when he spoke he had subdued his irritation.

"You would have a right to say and to think the worst of me if you thought me capable of such behaviour," he said, "but you wrong me grossly, Fred. Do you think I come here to steal—to take what isn't mine and to play with it, and then to run away like a coward? I come here as an honest man, who loves a good woman and hopes to win her."

"Then go away while there is time, before you compromise yourself or her—before you take a step that as a man of honour you can't back out of."

"Back out!" said Arthur hotly. "Upon my word, you have a mean enough opinion of me! Haven't I told you that it is my hope to win her, and that it will be my greatest happiness to succeed? Where is there a better or a sweeter girl, or a greater lady?"

"I grant you she is all you say—as good and beautiful and pure a girl as ever breathed—and fit to be the wife of a prince. You love her, perhaps, and if you married her you would always be tender to her, but you would have to marry her parents, too. Proudie is a very fine fellow. Nobody has a better right than I to know it, for he took me knowing what I was, and he treats me as if he had never heard my story; but, though he is as honest and generous a man as lives, he keeps a little eating-house, and he wears a white apron, and he eats with his knife. Mrs. Proudie was a cook, and superintended the kitchen department herself till she grew too infirm. She can't speak two words of correct grammar, or spell a letter as well as a child of four. Miss Betsy, though she can read and write and talk without mistakes, is no better than a respectable housekeeper, and has no wish to be. These would be your father and mother and aunt. You can't keep your wife shut up from them. She is too innocent and simple-minded and loyal to forsake them, even if you wished it of her, and how will you like daily association with them? How can you introduce them to your West End friends, or will you come here and settle in Gillespie Street, and expect them to come and see you? I entreat you to go while there is time, Arthur. You have done no harm yet. You are a gentleman, and you have taken no mean advantages. You can go without dishonour. I would have gone and hidden myself if that would have kept you away; but if I were to be lost to-morrow, you would come all the same."

"I've listened to you patiently," said Arthur at length, "and now it is your turn to listen to me. What you say is all true enough, and in many cases it would be the height of folly to disregard your advice; but my case is different. I have nobody to please but myself, and nobody to consult. As for my West End friends, as you call them, what have they done for me? Nellie has me to dinner when she is alone; Bellynge shows me plainly enough that I am not wanted when his fine friends are with him; I have found a kinder and an honester welcome here than in any house in Mayfair. I own that I would prefer a father-in-law with a little more culture, and a mother-in-law who didn't take all her ideas from the *Family Herald*, and who had a rather less gorgeous taste in dress; but one can't have everything. Proudie is an honest, guileless fellow, and if I became son to a duke I mightn't be able to say that of him. I never cared very much for the opinion of the world ——'

"No," interrupted Fred, "that is true; if you did you would not have known me; but, Arthur, you don't know till you come to the test how much we are guided by the world's notions and our own traditions. Let your friends be as careless as they please, they are refined, and have the manners of ladies and gentlemen, and the habits of decent society. The lack of these things galls terribly; you don't know how much a part of you they are till you miss them in your surroundings."

"Ah," said Arthur, "you have missed them?"

"No," Fred answered steadily, "I have forfeited the right to choose. These things seem small and insignificant enough to me in the wreck of my life, but it is different with you. You have a right to choose, and you should choose what you've been used to; it is safest in the end. I suppose a man has committed murder or suicide before now for nothing but for getting the wish of his heart and tying himself to illiterate, low-bred people."

"The girl I love can take her place with the highest in the land," said Arthur with a tender pride. "No one could whisper a word against her, and for the rest, I've chosen, and Delia, God bless her, if she will have me, will never have to blush for me when she sees me with her parents. I am free now. Ella has chosen somebody she likes better. I have money. If Delia consents we three will start for the new world together; if she refuses to leave her father and mother and sister, we will make a home here, and you will share it with us, old man."

Fred shook his head over this fond and foolish dream, but when he went upstairs he knew that his intervention had come too late.

The foregoing talk had taken place one wintry evening in the deserted library; Barbara and Delia, accompanied by George Tucker, were out at some little festival in the chapel where the family worshipped, and the older people were enjoying a well-earned repose in the parlour above.

"I'll go and shake hands with them," said Arthur, "you'll come?"

"I suppose I must," Fred said with a sigh and a reluctant look at the quiet corner by the stove.

As fortune would have it, the young girls came into the sitting room by one door, as the cousins entered it by the other.

The roses in Delia's cheeks burnt bravely when Arthur shook hands with her. She had on a little grey close bonnet, in which her sweet face was very fittingly framed—it had with the simplicity of her dress, an odd puritanic effect.

"I hope you have had a happy evening?" said Arthur.

"It was very pleasant," said Delia; "we had tea, and there were addresses and singing."

The young man looked at her. "I wish I had been there," he said. And he meant it, and yet it did not sound a very lively entertainment—a soiree in a dissenting chapel, with private refreshments in the shape of oranges and peppermints, no doubt.

"We do these things pretty well at Bethel. I'm a Presbyterian myself, Mr. Eastgate, but I say a good word for the Bethel folks when I can; they're liberal, and no mistake."

"Well, so you might," said Mrs. Proudie with a laugh;" a deal of the money comes out of your own pocket, Proudie."

"Hoots, woman, not a ha' penny too much," said Proudie stoutly. "Come Barbara, lass, tell us what ye did. Ye had a discoorse from Mr. Blatherwick?"

"Oh yes, two discourses. Mr. Eastgate would not have liked those," she smiled at Arthur frankly, "and Delia sang."

"You sing?" said Arthur in a low voice. "I have never heard you."

"She don't sing to command," growled the fierce little Tucker, who had been watching the direction of Arthur's glances, and saw something in them that fed his jealous fires.

"She will sing her bit 'spring' to Mr. Arthur if he wishes it," said Proudie in that tone of authority he used for the rebel Tucker. "And though I say it, Mr. Arthur, she's a grand hand at it; you'll go far or you hear a bonnier voice."

"You'll spoil the bairn with your havers, John Proudie," said Miss Betsy sternly. "Come, Delia, haste ye and sing your bit song; its long time you two lassies were in your beds, and Mr. Eastgate's waiting to go home."

Thus adjured, Delia lifted her hanging head. She looked rather shy and frightened, and Arthur said quickly—

"Please don't sing it if it distresses you. You are perhaps tired."

"No," said Delia simply. "I will try, but I don't sing well; I have never been taught."

She stood a little away from the others, her hands clasped, her little grey bonnet untied and hanging back by the strings, showing her sunny hair and round white throat.

She sang quite simply, and as the birds sing; but every note of that sweet, spontaneous music sank deep into Arthur's heart. It was a Scotch ballad with a sad minor refrain.

"I learnt her that," said her proud father, when the last faltering note died away; "my mother used to sing it."

"John Proudie," said the repressive Miss Betsy, "you've got no more voice than a corncrake. Delia, you go off to bed this instant."

Arthur took the hint and departed too; but the soft, sweet voice had nestled in his heart, and it went with him upon his way. *(To be continued.)*

